# Magnitude and Predictors of Anti-Retroviral Treatment (ART) Failure in Private Health Facilities in Addis Ababa, Ethiopia

**DOI:** 10.1371/journal.pone.0126026

**Published:** 2015-05-06

**Authors:** Yesunesh Teshome Yimer, Alemayehu Worku Yalew

**Affiliations:** 1 Clinton Health Access Initiative, Addis Ababa, Ethiopia; 2 School of Public Health, Addis Ababa University, Addis Ababa, Ethiopia; University of Ottawa, CANADA

## Abstract

**Background:**

The public health approach to antiretroviral treatment management encourages the public private partnership in resource limited countries like Ethiopia. As a result, some private health facilities are accredited to provide antiretroviral treatment free services. Evidence on magnitude and predictors of treatment failure are crucial for timely actions. However, there are few studies in this regard.

**Objective:**

To assess the magnitude and predictors of ART failure in private health facilities in Addis Ababa, Ethiopia.

**Methods:**

The study followed retrospective cohort design, with 525 adult antiretroviral treatment clients who started the treatment since October 2009 and have at least six months follow up until December 31, 2013. Kaplan Meier survival analysis and Cox proportional hazard model were used for analysis.

**Results:**

Treatment failure, using the three WHO antiretroviral treatment failure criteria, was 19.8%. The immunologic, clinical, and virologic failures were 15%, 6.3% and 1.3% respectively. The mean and median survival times in months were 41.17 with 95% Confidence Interval (CI) [39.69, 42.64] and 49.00, 95% CI [47.71, 50.29] respectively. The multivariate cox regression analysis showed years since HIV diagnosis (Adjusted Hazard Ratio (AHR)=13.87 with 95% CI [6.65, 28.92]), disclosure (AHR=0.59, 95% CI [0.36, 0.96]), WHO stage at start (AHR=1.84, 95% CI [1.16, 2.93]), weight at baseline (AHR=0.58, 95% CI [0.38, 0.89]), and functionality status at last visit (AHR=2.57, 95% CI [1.59, 4.15]) were independent predictors of treatment failure.

**Conclusion:**

The study showed that the treatment failure is high among the study subjects. The predictors for antiretroviral treatment failure were years since HIV diagnosis, weight at start, WHO stage at start, status at last visit and disclosure.

**Recommendations:**

Facilities need to monitor antiretroviral treatment clients to avoid disease progression and drug resistance.

## Introduction

Ethiopia is one of the low income countries experiencing high communicable, non-communicable disease burdens and injuries [1], including HIV/AIDS. As a response to the HIV/AIDS, Ethiopia has introduced the Antiretroviral Treatment (ART) program to save lives, to restore the mental and physical functions and to improve the quality of life of people living with HIV/AIDS [2]. As a result of public private partnership the country established, some private health facilities (Non-Government Organization (NGO) clinics and private hospitals) have started providing ART services since 2005 though the ART treatment outcomes in the private health facilities have not been well documented. ART needs to be regularly monitored for its outcomes: success or failure. Treatment failure in resource poor countries is determined by clinical, immunological and/or virological failures [3]. In such countries, patient monitoring system is dependent on clinic-immunological methods, though they lack sensitivity/specificity to detect virological failures of ART [4], which is the gold standard for ART treatment failure.

According to UNAIDS’s Country Progress Report on HIV/AIDS Response in Ethiopia in 2012, there were about 800,000 people who live with HIV in Ethiopia and among those who need antiretroviral treatment (ART) (with CD4 cutoff less than 200), only 86% were receiving it in 2011 [5]. The Ethiopian Public Health Institute estimated the number of ART needs in Ethiopia and Addis Ababa to increase by an average of 5% and 7% yearly from 383,969 and 45,135 in 2011 respectively [6]. On the other hand, the new WHO ART guideline, which changed the ART cutoff from CD4 count < = 200 to < = 350, is expected to increase the unmet need for ART [7]. As a result of this, the burden on the already overstretched public health facilities will increase and the public private partnership in ART program management will be of great help to counteract the overstretching public health facilities [8, 9]. In this regard, some private hospitals and NGO clinics have already been accredited to provide ART services.

Even if many HIV positive clients accessed ART, there was a treatment failure. Studies show that the magnitude of ART treatment failure in the public health sector in Ethiopia ranges from 20.4% [10] to 33.3% [11].

Some of the factors for ART treatment immunologic failure include:

- the degree of CD4T decline before and at the initiation of the treatment (the steeper the decline the steeper the rise),
- the rate of decline in viral-load,
- old age, co-infection, medications (ZDV, TDF+DDI), and
- persistent immune activation [4].

Another study in the public sector revealed that the risk factors for treatment failure include non-adherence and missed appointments [12].

On the other hand, the magnitude and predictors for ART treatment failure in the private sector have not been thoroughly assessed; there are only few studies on the assessment of the ART services in the private sector in Addis Ababa, Ethiopia, which discussed on predictors of immunologic failure and on retention of ART patients in care [11, 13]. The study has two objectives: determining the magnitude of treatment failure and identifying predictors of treatment failure among adults in the private health facilities in Addis Ababa. The study tried to speak to whether the nation-wide rapid ART scale-up in 2005 compromised access to quality treatment [14, 15]. Understanding the situation in the private facilities is important to prevent disease progression and avoid development of antiretroviral resistance.

## Materials and Methods

### Study area

The study was conducted in private facilities (not-for-profit) which are supported by PHSP. PHSP is a 5-year project (Sept. 2009-Sept. 2014), funded by the United States Agency for International Development (USAID). PHSP is the nation’s innovative and pilot project that demonstrated the feasibility of public—private partnerships in health [15]. PHSP supported 342 private health facilities, primarily in urban areas, in two city administrations and five regions (Addis Ababa and Dire Dawa City Administrations, Tigray, Amhara, Oromia, Southern Nation, Nationalities, and People (SNNP), and Harari regions) of Ethiopia to enable and provide major public health services: Public- Private Mix-Directly Observed Therapy Short-Course (PPM DOTS), comprehensive HIV care (HIV Testing and Counseling (HTC), Prevention of Mother-to-Child Transmission (PMTCT), and ART), family planning, malaria and STIs.

The ART program is implemented in 20 health facilities (10 private hospitals and 10 NGO clinics/health centers) in the five regions/city administrations with the exception of SNNP and Oromia. The not-for-profit health facilities provide free treatment; however, patients pay out of pocket for health services provided by for-profit health facilities except for ART drugs [15]. Since eighty percent of the ART clients in PHSP supported facilities are found in Addis Ababa (including those that recently joined PHSP, World Wide Orphans), this study focused on the five NGO clinics/health centers (Mekdim Ethiopia Clinic, Missionary of Charity Clinic, St. Gabriel Catholic Health Center, Selam Children’s Village Clinic, and African Services Committee Clinic) in Addis Ababa City Administration. These facilities have registered data on at least six months of follow up of ART clients.

In 2013, 6 public hospitals, 37 Government health centers, 14 private hospitals, and 9 Non-Government clinics provided ART services in Addis Ababa. According to the annual report of the Addis Ababa City Administration Health Bureau for July 2012 to June 2013, as of July 7, 2013, there were a total of 125,994 HIV positive clients (94.4% adults) who ever enrolled to ART services, among which 74,986 (94.8% adults) had ever started ART and there were 53,677 (95.5% adults) currently on ART. The contribution of the private hospitals and NGO clinics to the ever enrolled, ever started ART and currently on ART was 20,159 (16.0%), 16,572 (22.1%) and 11,165 (20.8%) respectively. From NGO contributions, the five NGO facilities that are supported by the Private Health Sector Program (PHSP) in Addis Ababa had contributed 36.2%, 38.9%, and 44.5% to the ever enrolled, ever started and currently on ART, respectively.

### Study design

A retrospective cohort study design was employed to retrieve relevant information from the registration books to address the objectives of the study Those HIV patients who are 18 years and above and who started ART since October 2009, after PHSP started supporting the program, and who had at least six month follow up until December 31, 2013 were included in this study.

### Sample size

The sample size of this study was determined considering the requirements for the two objectives. The maximum of the two sample sizes was taken as the total sample size. For the first objective, single proportion sample size determination and for the second objective, sample size for two proportion techniques wereadopted. Epi-info version 3.5.4 was used to calculate the sample sizes.

For the first objective, as there was no information on the magnitude of treatment failure among adults in the private health facilities, a 50% prevalence was assumed Moreover, a 3% margin of error (to increase the precision of the estimate), and 95% confidence level were assumed. After adjusting for the finite population correction (total HIV patient population who are currently on ART in the private facilities was 653), the sample size required for the first objective was 406.

For the second objective, two proportions formula was used. Among the different predictors of treatment failure, baseline CD4 count< 200 cells/mm^3^ yield the maximum sample size. A power of 80%, a 5% level of significance and ratio of 1:2 (exposed: unexposed) were assumed. Moreover, a 29.9% treatment failure among patients with baseline CD4 count less than 200 cells/mm^3^ (exposure) and a 15.7% treatment failure among patients with baseline CD4 count≥ 200 cells/mm^3^ were assumed [12].

Based on the above assumptions, it was found that the sample size required for addressing the second objective as 109 exposed and 218 non-exposed, a total of 327 patients on ART. However, to increase the precision of the estimates and power of the study, the investigators decide to include all adult ART clients in the study area, i.e., 525 patients who fulfilled the inclusion criteria.

### Data collection tools and procedures

The data collection tool, prepared in English, was a data abstraction form containing the most relevant variables in relation to ART treatment outcomes as described in different studies. Data were collected both from patient follow up form and the electronic database for ART program (Register Database) which captured data on different variables of patient demographic and treatment information from his/her patient card, follow up card and ART register.

The data was retrieved by five data clerks that have diploma and experience in managing ART data at each facility. They received training and the principal investigator made a close supervision to ensure the data quality. The data had no any personal identifier like names and addresses except ART codes given to each patient. The data were abstracted from Nov. 15, 2013—Dec. 31, 2013. It included those records from Oct.1, 2009—Dec.31, 2013.

### Operational definitions

❖ Immunological failure:- Fall of CD4 count to baseline (or below) OR 50% fall from on-treatment peak value OR Persistent CD4 levels below 100 cells/mm^3^ [16].
❖ Clinical failure:- New or recurrent WHO stage 4 condition OR new or recurrent WHO stage 3 with pulmonary TB and/or severe bacterial infections [16].
❖ Virologic failure:- When the plasma viral load is above 10000 copies/ml, OR, when an HIV RNA level >200 copies/mL [17].
❖ Treatment failure is considered in this study, if a patient has either of the clinical, immunological or virologic failure [16].
❖ Poor Adherence is defined in terms of total missed appointments described by greater than three days per month [18].
❖ The time to detection of treatment failure:- the time between ART initiation and detection of failure of first line ART [18].

### Data Quality

The data collected was entered first into EPI Info Version 3.5.4 and then exported to SPSS version 20 software. It was cleaned for data encryption and logical errors by the principal investigator. As the study used secondary data from facility registers and follow up charts, there were missing values in many variables. In order to address the missing values of some variables like CD4 counts and weight, imputation technique was applied using SPSS.

### Variables

#### Dependent variables

Antiretroviral treatment failure and time to the occurrence of treatment failure.

#### Independent variables

Age, sex, marital status, education, HIV disclosure status, years since HIV diagnosis, type of regimen, weight at baseline, CD4 at baseline, functional status at baseline, WHO stage at baseline, status at last visit, drug substitution, TB treatment, adherence in terms of 3 days missed appointments per month, and number of episodes of poor adherence.

### Data Analysis

Univariate and bivariate analyses have been done to see the magnitude of the different characteristics of the study subjects. Kaplan Meier survival analysis and Cox proportional hazard model were used to describe the time to treatment failure and to assess independent predictors of the first line ART treatment failure respectively. SPSS version 20 and STATA Version 12.1 software was used for the analysis.

### Ethical Statement

The study was based on secondary data already gathered by Abt Associates Inc./Private Health Sector Program for many years. There was no any contact with individual patients to get informed consent. Abt Associates Inc./Private Health Sector Program and the private health facilities, who are owner of the data, gave informed verbal consent. But written ethical approval was obtained from University of Gondar and Addis Ababa City Administration Health Bureau. Since it was not a primary data, rather a secondary data, there were no chances to contact the patients to get written consent. The already documented data were retrieved. The ethics committees have approved all the methodological and ethical procedures. The data collected bore no specific identification of clients on ART. The information is kept confidential. Only the investigators have access to the data set. The patient records were anonymized and de-identified prior to analysis. The Addis Ababa City Administration Health Bureau’s ethical clearance letter was dated Dec. 11, 2013 with reference # AAHB/2172/227.

## Results

### Socio-demographic and clinical characteristics

Among the 525 study subjects, 354 (67.4%) were female. The mean (±SD) age at start of ART was 33.58 (±9.47) and the median age was 32 (IQR: 27.00 – 38.00), where most of the study subjects (80.6%) fell in the age group 25–49. Most of the study subjects had primary education (45.5%) and those with secondary and above education level accounted to 30.2%. Thirty-four point one percent of them were married while 13.14% were single. The minimum follow up time was six months while the maximum was 50 months. The median stay on ART in the health facilities was 21 months (IQR: 11.00 – 36.00).

Most (44.2%) of the study subjects were on TDF/3TC/EFV at baseline. The next most widely used baseline regimens were AZT/3TC/NVP (21.3%) and AZT/3TC/EFV (13%). The median CD4 count at baseline was 177 cells/mm^3^ (IQR: 114.00 – 248.00). Table 1 shows the baseline characteristics of the study subjects. The descriptive statistics on major variables are also depicted in Table 2.

**Table 1:** Socio demographic and clinical characteristics of ART patients in private health facilities in Addis Ababa, 2009–2013.

Characteristics	Number (valid %)
Age (in years)	
18–24	64 (12.2%)
25–49	423 (80.6%)
>50–64	38 (7.2%)
Sex	
Female	354 (67.4%)
Male	171 (32.6%)
Time since HIV diagnosis	
< 1 year	77 (14.7%)
1–2 years	250 (47.6%)
2–3 years	83 (15.8%)
3–4 years	49 (9.3%)
> 4 years	66 (12.6%)
Functional status	
Working	423 (80.6%)
Ambulatory	64 (12.2%)
Bedridden	38 (7.2%)
WHO stage	
I	116 (22.1%)
II	167 (31.8%)
III	193 (36.8%)
IV	49 (9.3%)
CD4 Count (cells/mm^3^)	
0–49	42 (8.0%)
50–99	72 (13.7%)
100–199	188 (35.8%)
200–349	204 (38.9%)
350–999	19 (3.6%)
Regimen started	
TDF-3TC-EFV	214 (44.2%)
AZT-3TC-NVP	103 (21.3%)
AZT-3TC-EFV	63 (13.0%)
ZDV-3TC-NVP	39 (8.1%)
d4T-3TC-NVP	20 (4.1%)
d4T-3TC-EFV	19 (3.9%)
ZDV-3TC-EFV	12 (2.5%)
TDF-3TC-NVP	6 (1.2%)
OTHER	8(1.7%)
Weight	
> 50 KG	262 (52.1%)
< 50 KG	241 (47.9%)

**Table 2:** Descriptive Statistics of characteristics of ART patients in private health facilities in Addis Ababa, 2009–2013.

	N	Minimum	Maximum	Median	Inter quartile range	Mean	Standard error of the mean	Std. Deviation
Age	525	18	76	32	11	33.59	0.413	9.472
Weight at Baseline	503	19.02	94	51	13	51.88	0.447	10.03
Episode of Poor or fair Adherence over the follow up period	484	0	4	0	0	0.14	0.022	0.475
CD4 count at Baseline	525	5	768	177	134	189.27	4.811	110.245
Years alive since HIV diagnosis	525	0	15	2	2	2.42	0.103	2.363
Total missed appoints in days over the follow up time	208	0	120	0	1	12.28	1.683	24.273
Calculated months on ART	525	6	50	21	25	23.43	0.608	13.929

### Treatment failure

Among the 525 study subjects, 80.2% were right censored (free of treatment failure). It was found that a total of 104 subjects (19.8%) have indication of treatment failure. Of the 11 persons who had a viral load test, 7 were confirmed as treatment failure, which results in a virologic failure of 1.3% from the total. The immunologic failure was found to be 15% (79 cases failed immunologically), while the clinical failure was 6.3% (33 cases). There were 7 cases (1.3%) with both virological and immunological failure. The number of cases who failed immunologically and clinically was 8 (1.5%) while there were no cases who failed both clinically and virologically. Among the patients who failed virologically, 3 (42.8%) were transfers out, 1 (14.3%) dead and 3 (42.8%) drop outs. From the 104 treatment failure, 70 (67.3%) were still alive and on ART first line regimen, while 20 (19.2%) were transfer outs; drop outs and death accounted for 7 (6.7%) each.

### Predictors of treatment failure

Among the 525 study subjects, 302 (57.5%) had baseline CD4 count less than 200 cells/mm^3^. The mean and median CD4 counts were 189.26 cells/mm^3^ (SD = ±110.46) and 177 cells/mm^3^ (IRQ: 114.00 – 248.00) respectively. Those who started ART at WHO stage III and IV accounted for 242 (46.1%). Ten point five percent of the study subjects had shown one and more than one episodes of poor adherence, whereas in terms of number of days of missed appointments, 20.2% had missed treatment for more than 15 days.

**Fig 1:**
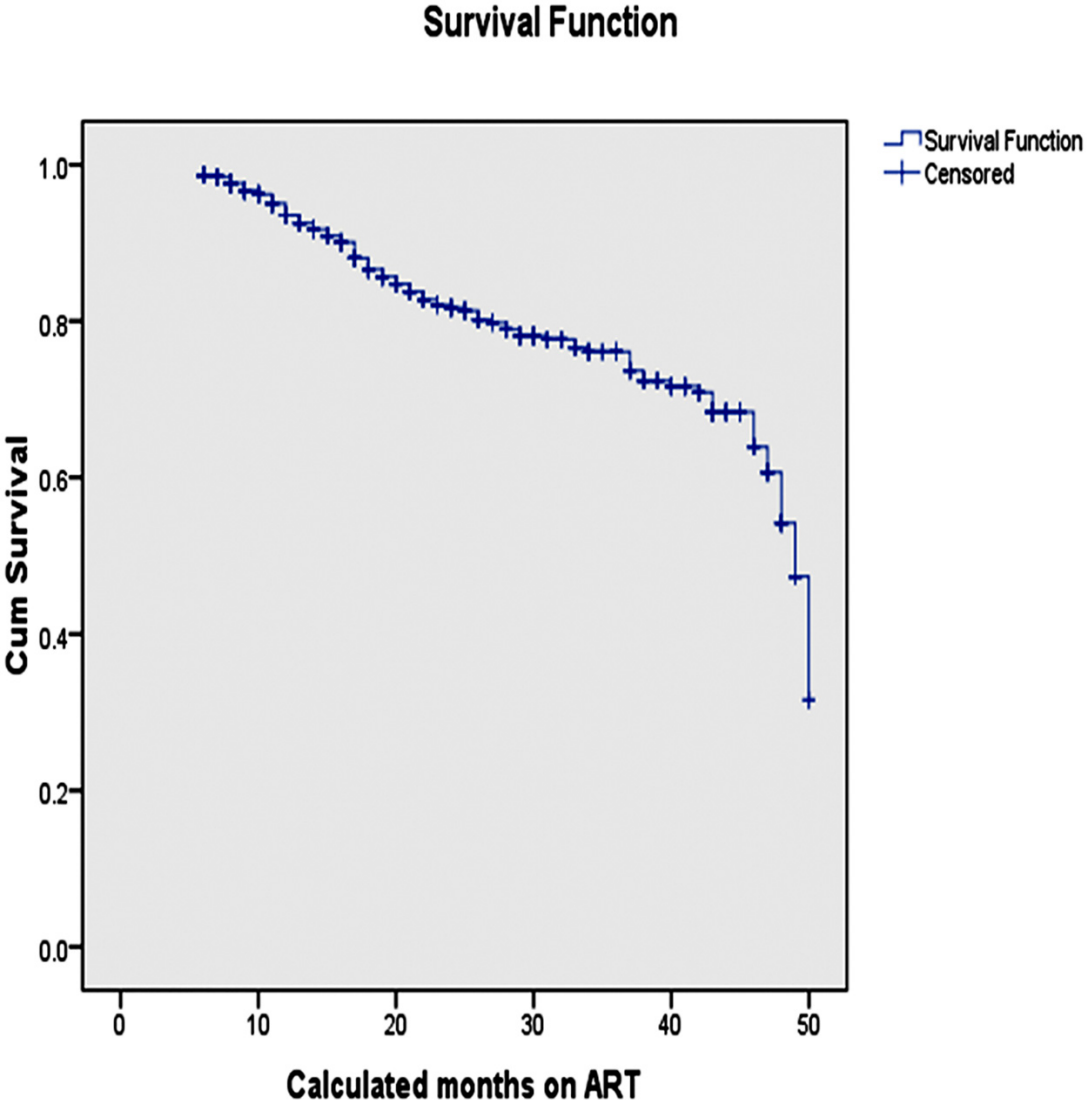
The Kaplan Meier Survival function of ART patients in private health facilities in Addis Ababa, 2009–2013.

Using the Kaplan Meier method, the mean survival time (the expected time to event) was found to be 41.17 months with 95% CI (39.69, 42.65) while the median survival time was 49.00 months with 95% CI (47.706, 50.294) (Fig 1). Survival in this study means no treatment failure. The treatment failure incidence rate was 8.45 per 1000 person-months (Table 3). Table 4 shows the socio demographic variables as predictors of first line treatment failure.

**Table 3:** Person-time contributed by study subjects by status at the last visit in the private health facilities in Addis Ababa, 2009–2013.

Status category	Person-time in months	Treatment Failures	Treatment Failure incidence rate	95% Confidence Interval	
				lower	higher
Alive on ART	10329	70	0.006777	0.005362	0.008566
Transfer out	1123	20	0.017809	0.01149	0.027605
Dead	240	7	0.029167	0.013905	0.06118
Drop	474	7	0.014768	0.00704	0.030977
Lost	134	0	0	-	-
**Total**	**12300**	**104**	**0.008455**	**0.006977**	**0.010247**

**Table 4:** Socio demographic characteristics as predictors of first line ART treatment failure in adults in private health facilities in Addis Ababa, 2009–2013.

Covariate	Treatment failure (%)	Free of Treatment failure (%)	Crude hazard ratio (95% CI)
Age (in years)			
18–24	14 (21.9%)	50 (78.1%)	1
25–49	81 (19.1%)	342 (80.9%)	0.808 (0.458,1.426)
50–64	7 (22.6%)	24 (77.4%)	1.239(0.499,3.075)
> 65	2 (28.6%)	5 (71.4%)	1.063 (0.241, 4.687)
Sex			
Female	65 (18.4%)	289 (81.6%)	1
male	39 (22.8%)	132 (77.2%)	1.247 (0.838, 1.855)
Marital status			
single	15 (21.7%)	54 (78.3%)	1.693 (0.739, 3.879)
married	33 (18.4%)	146 (81.6%)	1.487 (0.709, 3.116)
divorced	19 (18.3%)	85 (81.7%)	1.517 (0.686, 3.357)
widowed	14 (20.9%)	53 (79.1%)	1.324 (0.572, 3.061)
partner	9 (17.0%)	44 (83.0%)	1
Education			
illiterate	14 (13.7%)	88(86.3%)	1
primary	34 (17.8%)	157 (82.2%)	1.521(0.815, 2.839)
secondary	27 (27.8%)	70 (72.2%)	2.181(1.137, 4.182)
tertiary	3 (10%)	27 (90%)	0.931 (0.266, 3.257)
Disclosure			
Disclosed	79 (22.8%)	268 (77.2%)	1.529 (0.935, 2.500)
Not disclosed	5 (11.4%)	39 (88.6%)	0.984 (0.368, 2.631)
Not known	20(14.9%)	114 (85.1%)	1

All the sub groups for the above socio demographic variables showed no significant hazard ratios from the bivariate cox hazard ratio analysis. On the other hand, the clinical characteristics such as the weight at baseline, WHO stage at start, functional status at baseline, drug substitution, missed appointments as indicators of poor adherence, status at last visit, drug substitution, disclosure and years alive since HIV diagnosis had hazard ratios with p<0.2.

### The comparison of survival functions

**Fig 2:**
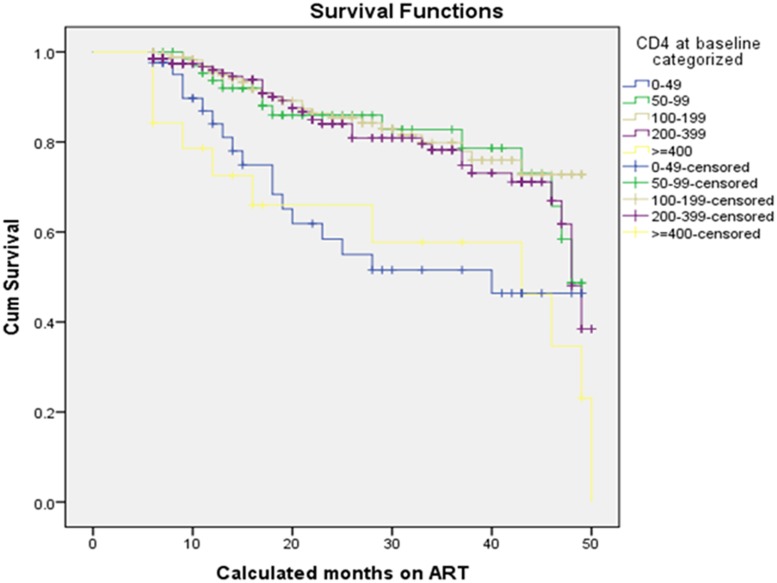
Survival functions of different groups of CD4 at baseline of ART patients in private health facilities in Addis Ababa, 2009–2013.

**Fig 3:**
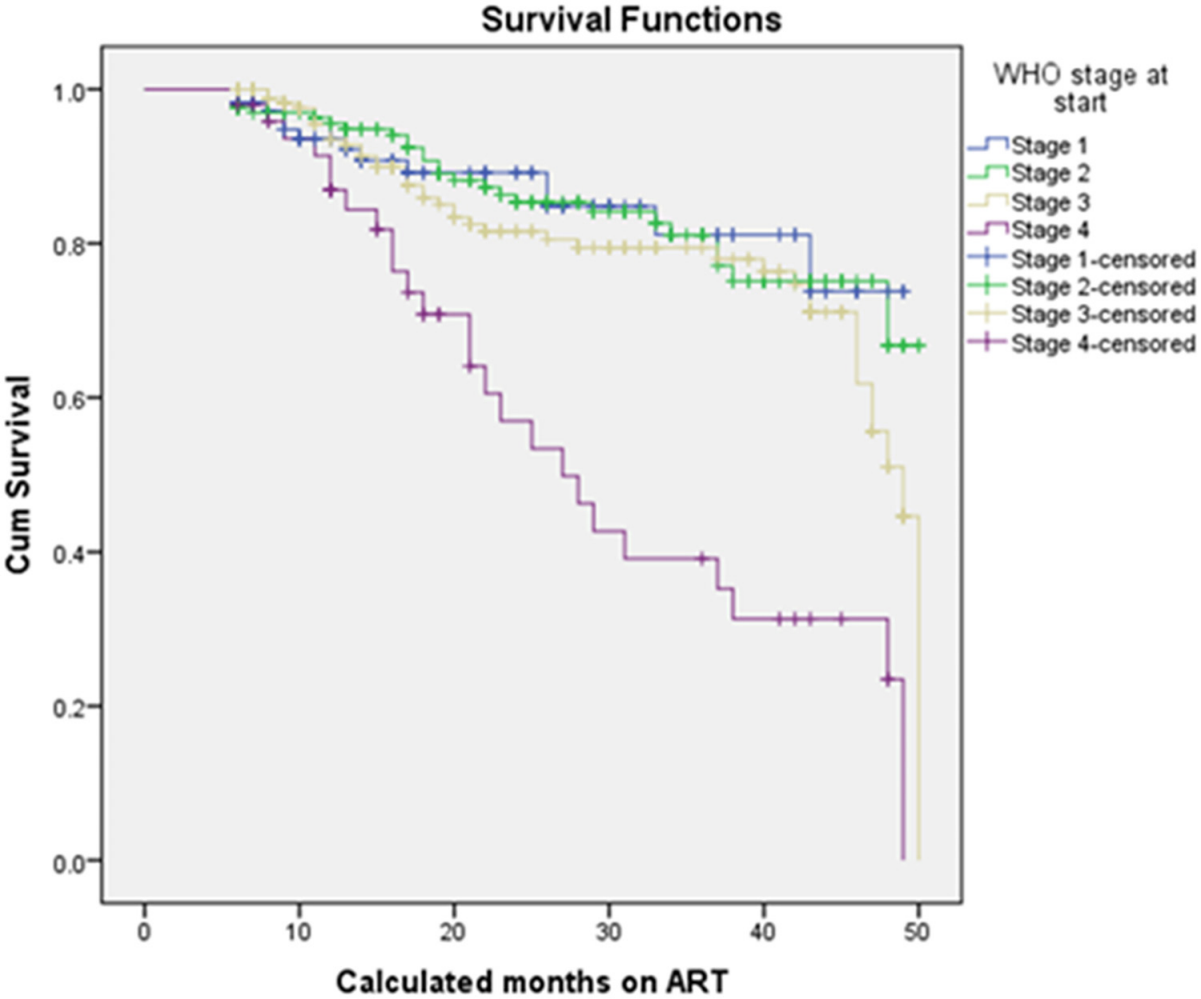
Survival functions of different groups of WHO stage at start of ART patients in private health facilities in Addis Ababa, 2009 – 2013.

**Fig 4:**
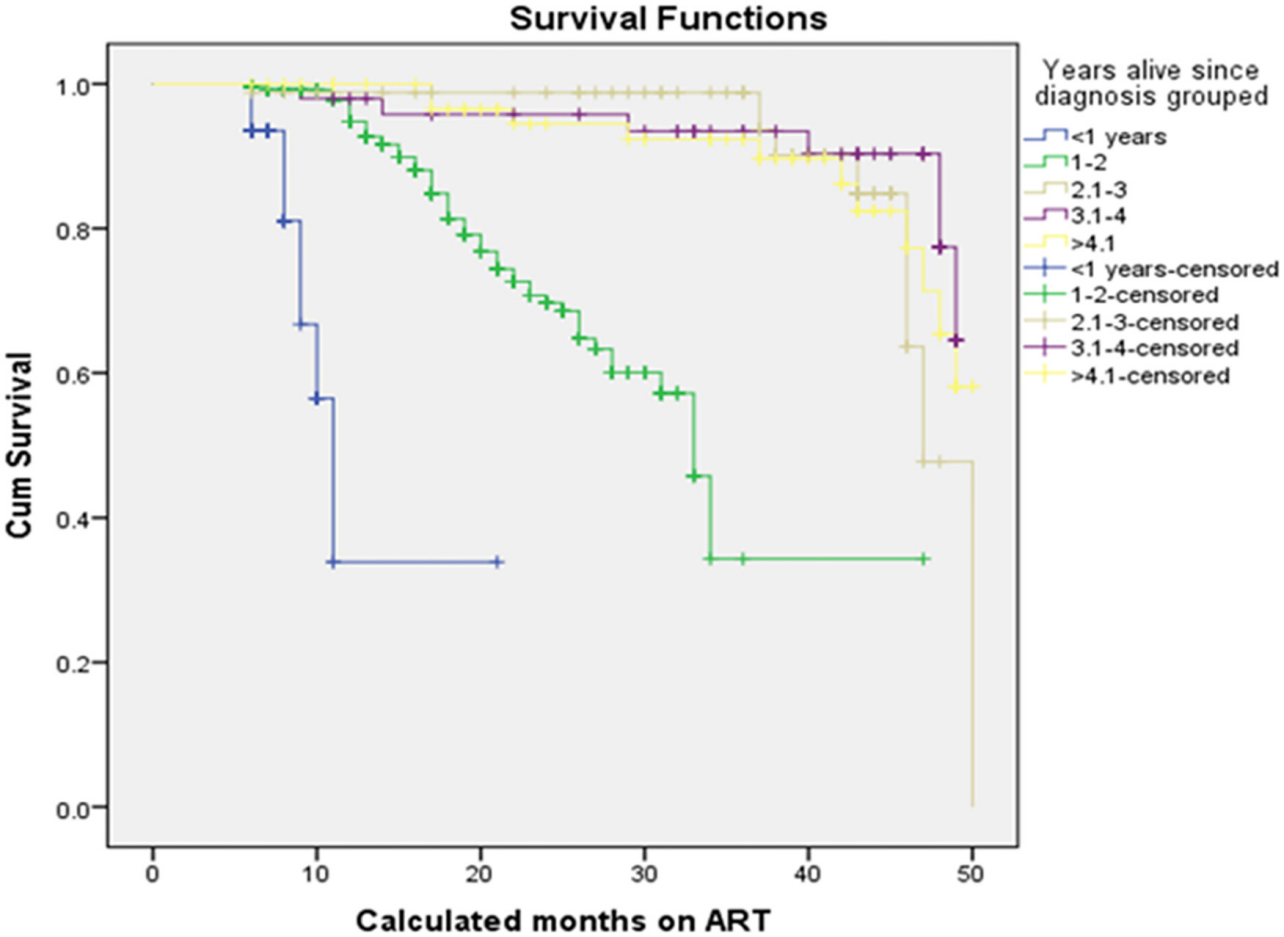
Survival functions of different groups of Years alive since HIV diagnosis of ART patients in private health facilities in Addis Ababa, 2009 – 2013.

**Fig 5:**
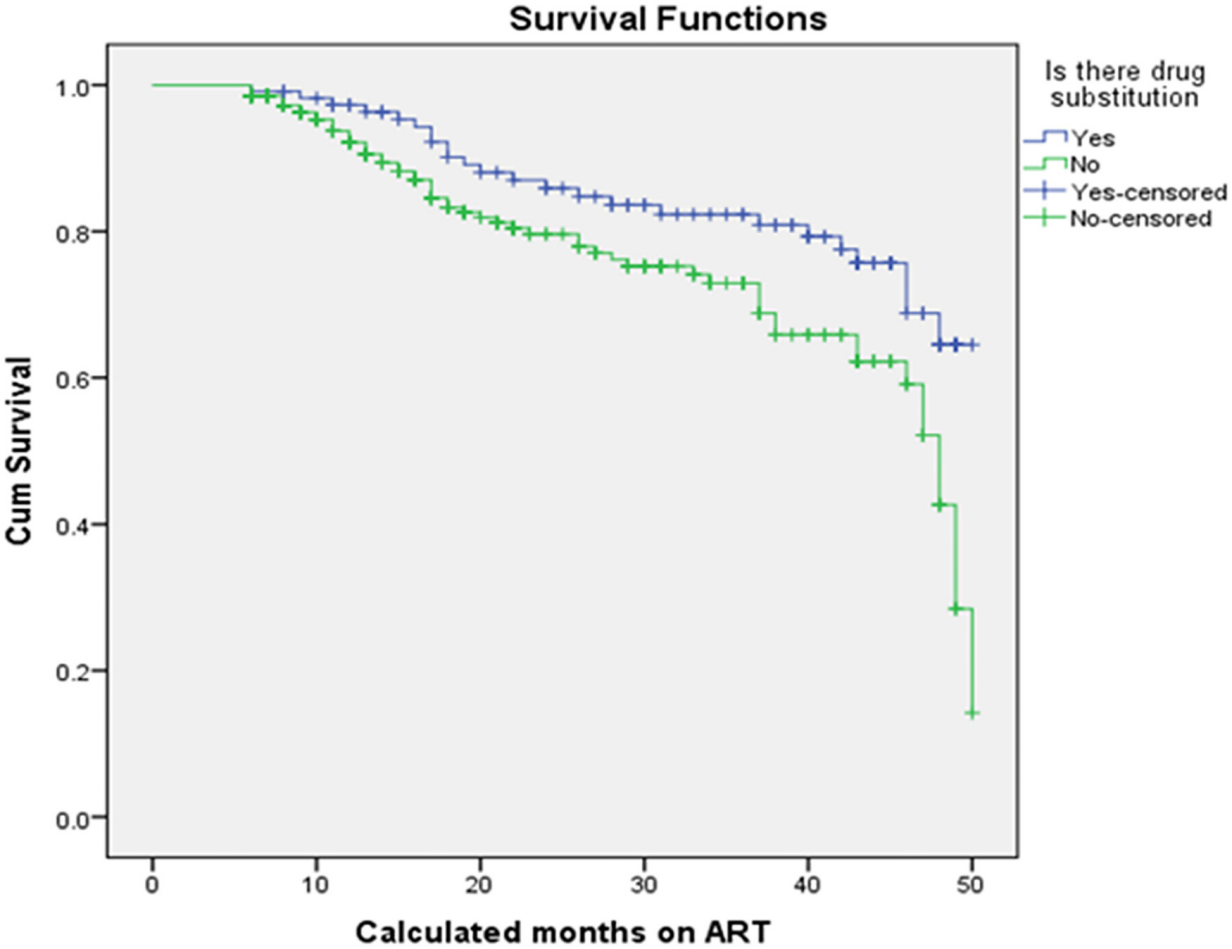
Survival functions of different groups of drug substitution for ART patients in private health facilities in Addis Ababa, 2009 – 2013.

The Log rank test (Mantel-cox) showed that there was significant difference in survival times between the different categories of adherence defined in terms of 3 missed appointments per month (p<0.001), drug substitution (p = 0.018), status at last visit (p<0.001) and years since HIV diagnosis (p<0.001). In addition to these, differences in survival times among categories of CD4 at baseline (p<0.001), the WHO stage at baseline (p<0.001) and TB treatment (p<0.001) were observed. Table 5 and Figs 2, 3, 4, and 5 show the differences. On the other hand, no significant difference in survival times was observed among groups of gender, age, marital status, educational status, weight at baseline, disclosure status, regimen, and baseline functional status.

**Table 5:** Means and Medians for Survival Times for groups of different variables on ART patients in private health facilities in Addis Ababa, 2009–2013.

Variables	Mean	Std. Error of the mean	95% Confidence Interval of the mean		Median	Std. Error of the median	95% Confidence Interval of the median	
			Lower Bound	Upper Bound			Lower Bound	Upper Bound
TB treatment after 6 month on ART								
No	41.923	0.76	40.433	43.413	50	1.201	47.645	52.355
Yes	31.931	2.847	26.351	37.51	33	6.021	21.198	44.802
Overall	41.17	0.753	39.694	42.645	49	0.66	47.706	50.294
Is there drug substitution								
Yes	43.505	1.236	41.082	45.929	.	.	.	.
No	39.517	1.147	37.268	41.766	48	0.999	46.042	49.958
Overall	41.114	0.861	39.428	42.801	49	0.679	47.67	50.33
CD4 at baseline categorized (cells/mm^3^)								
0–49	32.612	2.909	26.91	38.314	40	.	.	.
50–99	42.116	1.732	38.72	45.511	48	.	.	.
100–199	42.485	1.142	40.247	44.723	.	.	.	.
200–399	41.88	1.162	39.603	44.158	48	0.889	46.258	49.742
> = 400	33.012	4.612	23.973	42.051	43	11.269	20.913	65.087
Overall	41.17	0.753	39.694	42.645	49	0.66	47.706	50.294
Years alive since HIV diagnosis grouped								
<1 years	13.301	1.489	10.382	16.22	11	0.611	9.802	12.198
1–2 years	32.436	1.812	28.885	35.987	33	1.102	30.84	35.16
2.1–3 years	46.684	0.927	44.867	48.502	47	1.076	44.891	49.109
3.1–4 years	46.545	1.288	44.021	49.07	.	.	.	.
>4.1 years	46.361	1.125	44.155	48.567	.	.	.	.
Overall	41.17	0.753	39.694	42.645	49	0.66	47.706	50.294

The bivariate cox proportional hazards analysis showed WHO stage at baseline (p = 0.003), regimen substitution (p = 0.020), years since HIV diagnosis (p<0.001), total days of missed appointments,3, per month (p<0.001), baseline functional status (p = 0.075), CD4 count at baseline with cutoff point 50 cells/mm^3^ (p = 0.002), disclosure (p = 0.063), baseline weight (p = 0.235) and status at last visit (p<001) had significant relationship with treatment failure as predictors. Entering these into the multivariate cox regression analysis gave five significant predictors (status at last visit, disclosure, years since HIV diagnosis, weight at baseline, and WHO stage at baseline). The adjusted hazard ratios of years alive since HIV diagnosis, weight at baseline, WHO stage at start, status at last visit, and disclosure were found to be 13.87 (95% CI [6.65,28.92]), 0.58 (95% CI [0.38,0.89]), 1.84 (95% CI [1.16,2.63]), 2.57 (95% CI [1.59,4.15]), and 0.59(95% CI [0.36,0.96]) respectively (see Table 6).Those who had been less than three years since HIV diagnosis had a 13.87 times more hazards towards treatment failure when compared to those who were diagnosed for HIV more than three years. Those who started ART at WHO stage 3 or 4 had a 1.84 times more hazards towards treatment failure when compared to those who started at WHO stage 1 or 2. Those with status at last visit other than ‘Alive’ had a 2.57 times more hazards towards treatment failure when compared to those who are ‘Alive’ on ART. On the other hand, non-disclosure had been found to be a protective factor against treatment failure. Likewise, having weight at baseline less than 50 kg had been a protective factor against treatment failure.

**Table 6:** Multivariate analysis showing predictors of ART treatment failure in private health facilities in Addis Ababa, 2009–2013.

Covariate	Treatment failure (%)	Free of Treatment failure (%)	Crude hazard risk (95% CI)	Adjusted hazard ratio (95% CI)
WHO clinical stage				
Stage 1 or 2	39 (13.8%)	244 (86.2%)	1	
Stage 3 or 4	65 (26.9%)	177 (73.1%)	1.845 (1.239, 2.749)	1.844 (1.160, 2.931)
Adherence				
Poor adherence (missed appointment greater than 3 days per month)	6(50%)	6(50%)	5.528 (2.395, 12.762)	2.145 (0.870,5.293)
Good adherence (missed appointment less than 3 days per month)	98(19.1%)	415(80.9%)	1	
HAART Drug substitution				
Yes	24 (21.2%)	89 (78.8%)	1	
No	80(19.4%)	332(80.6%)	1.733 (1.089, 2.760)	1.122 (0.677, 1.860)
CD4 count at initiation (cells/mm^3^)				
<50	17 (40.5%)	25 (59.5%)	2.287 (1.359, 3.851)	0.635 (0.361, 1.120)
> = 50	87 (18.0%)	396 (82.0%)	1	
Baseline functional status				
Working	73 (17.3%)	350 (82.7%)	1	
Not working	31 (30.4%)	71 (69.6%)	1.468 (0.961, 2.243)	1.416 (0.857, 2.339)
Weight at baseline				
Less than 50 kg	45 (20.6%)	196 (79.4%)	0.785 (0.526, 1.171)	0.581 (0.381, 0.887)
Greater than 50 kg	54 (18.7%)	208 (81.3%)	1	
Status at last visit				
Alive on ART	70 (16.9%)	343 (83.1%)	1	
Others	34 (30.4%)	78 (69.6%)	3.358 (2.191, 5.148)	2.572 (1.593, 4.153)
Years since HIV diagnosis				
< = 3 years	74 (22.6%)	253(77.4%)	12.039 (6.44, 22.504)	13.868 (6.65, 28.92)
> 3 years	30(15.2%)	168(84.8%)	1	
Disclosure				
not disclosed	25 (14.0%)	153 (86.0%)	0.652 (0.415,1.023)	0.585 (0.358, 0.955)
disclosed	79 (22.8%)	268 (77.2%)	1	

## Discussion

This study was designed to show the magnitude and predictors of ART treatment failure in private health facilities in Addis Ababa, Ethiopia. The ART program is one of the programs provided through public-private partnership (PPP). The PPP enabled 16 private health hospitals (from which two had terminated providing the service) and 9 NGO clinics/health centers in Addis Ababa. PPP is believed to be a scheme to deliver different health care services through a cost effective way reducing a significant burden from the public health sector [19–21].

The Private Health Sector Program is a USAID funded project being implemented by Abt Associates Inc. in Ethiopia. It is a pioneer project which is working to ensure PPP in major public health service delivery (TB, HCT, ART, PMTCT, Malaria, Family Planning, and STI). The ART program, through PPP, started in 2009 and is currently being provided in 20 private and NGO health facilities.

### Magnitude of treatment failure

The overall treatment failure is found to be 19.8% with incidence rate of 8.46 failures per 1000 person-months. Even though the overall treatment failure, is in line with the magnitude of treatment failures in resource limited settings [22–24] and as the sensitivity and specificity of immunologic and clinical failures were discussed to be low [25–32], it has to be further accompanied by viral load tests to confirm true treatment failure and to take optimal decision of switching and avoiding misclassification [23, 33].

Though viral load testing is very limited in low income countries, it has confirmed treatment failure for many of those suspected of failure. From those suspected, 11 patients with viral load tested, seven of them were confirmed as having treatment failure (63.7%). The treatment failure magnitudes computed using all, two or one of the three criteria, alert that there should be cautious and frequent monitoring and evaluation of the ART treatment outcomes by health care providers.

Unlike the findings from a study conducted in Kenya [26], where the clinical failure exceeded the virological and immunological failures, the results from this study showed that the magnitude of treatment failure as defined by immunological criteria was high (15%); followed by the clinical failure (6.3%) and by virologic failure (1.3%).

Most of the failed cases were those who were in age groups 25–49 (77.9%), female (62.5%), married (36.7%), had primary education (43.6%), and disclosed their HIV status (76%), which is against a finding from a study in Tanzania [34]. The high proportion of treatment failure in disclosed patients could be fear of the stigma because of the disclosure and as a result of non-adherence. From the clinical characteristics, it was observed 62.5% of the failures were those in WHO stage 3 or 4 at start, 98.9% in NRTI+NNRTI regimen base, 71.1% who had no drug substitution, 100% who had viral load >10000 cells/ml, 70.2% who were working, 71.2% with three or less years since HIV diagnosis, and 67.3% with status alive and on ART. This is in line with the findings from past studies in resource limited settings [26, 34]. On the contrary, 83.7% who had CD4 count greater than 50 cells/mm^3^ and 54.5% those who had weight greater than 50 KG tend to fail on their ART treatment. These could be due to other confounding factors not captured in the study and because of the small sample size used in the study. In the descriptive analysis, even though 94.2% with good adherence seemed to fail, the multivariate analysis showed those with poor adherence had a 2.145 times more risk of failure compared with those with good adherence, though not significant.

### Predictors of treatment failure

Even though the bivariate cox hazard analyses gave significant crude hazards of years alive since HIV diagnosis, CD4 count with cutoff point 50 cells/mm^3^, missed appointments with cutoff point 3 days per month, drug (HAART) substitution, baseline functional status, weight at baseline, WHO stage at baseline, status at last visit, and disclosure status, the multivariate cox analysis identified five significant predictors of treatment failure; which were years since HIV diagnosis, disclosure, WHO stage at start, weight at baseline, and status at last visit. The findings from years since HIV diagnosis, WHO stage at start and status at last visit are in line with findings from a study done in Urban HIV Clinic [12, 35]. A study done in Mozambique showed that starting ART at later stages of WHO (III and IV) had a risk of poor outcomes and as a result it recommended initiation of ART at early stages [35]. On the other hand, the disclosure and weight at baseline findings are against the findings from a study in Tanzania [34]. These could be due to the non-adherence as a result of stigma and discrimination after disclosure and the perception that those with higher weights than 50 kg do not need to stick to prescriptions.

Identifying the predictors helps to suspect treatment failure and for intervention. Especially, earlier detection of virological failure allows both targeted adherence interventions and better preservation of efficacy of second-line regimens [16].

#### Limitation

The study used secondary data which are usually not complete. As a result, some variables have missing data which they are left from the analysis while they may affect the outcome variable. Besides, some important behavioral and contextual factors were not captured in the ART registers and/or follow up charts and electronic databases which the study drew data from.

## Conclusion and Recommendations

The private health facilities under this study showed a treatment failure of 19.8%, immunologic failure 15%, clinical failure 6.3%, and virologic failure of 1.3%. The failure rate is comparable to that on similar settings. The immunologic failure can be minimized by closely following the patients. The failures should be confirmed by the gold standard, viral load test, before switching. The facilities can use the viral load test freely through the PPP.

The significant predictors for ART treatment failure were years since HIV diagnosis, weight at start, WHO stage at start, status at last visit, and disclosure.
